# Micro-costing analysis of a community-based psychiatric intervention among people who inject drugs in Haiphong, Vietnam

**DOI:** 10.3389/fpsyt.2025.1676340

**Published:** 2025-12-04

**Authors:** Philippe Trouiller, Sao Mai Le, Giang Hoang Thi, Huong Duong Thi, Oanh Khuat Thi Hai, Tuyet Thanh Nham Thi, Jean-Pierre Moles, Didier Laureillard, Don C. Des Jarlais, Nicolas Nagot, Laurent Michel, Nathalie Pelletier-Fleury

**Affiliations:** 1 Center for Research in Epidemiology and Population Health, Inserm Unité Mixte de Recherche (UMR) 1018, Paris Saclay University, Versailles Saint Quentin University, Villejuif, France; 2 Pierre Nicole Center, French Red Cross, Paris, France; 3 Hai Phong University of Medicine and Pharmacy, Hai Phong, Vietnam; 4 Supporting Community Development Initiatives, Hanoi, Vietnam; 5 Pathogenesis and Control of Chronic and Emerging Infections, University of Montpellier, Inserm, University of Antilles, Montpellier, France; 6 Infectious Diseases Department, Caremeau University Hospital, Nîmes, France; 7 College of Global Public Health, New York University, New York, NY, United States

**Keywords:** mental health, people who inject drugs, community-based intervention, micro-costing, peers

## Abstract

**Introduction:**

Community-based interventions and peer-support have been shown to improve health outcomes and are increasingly promoted in mental health care. To guide further policy makers, we aimed to estimate, from a societal perspective, the cost of a community based psychiatric intervention for people who inject drugs (PWID).

**Methods:**

From March 2022 to May 2022 in Hai Phong, Vietnam, PWID currently or previously diagnosed with a psychiatric disorder were recruited in a 12 months follow-up cohort and received psychiatric and harm-reduction services supported by peers (Drive-Mind 2 cohort study, NCT05886504). Using a micro-costing approach, we estimated the annual per-participant cost for screening PWID for psychiatric disorders, and for providing them for follow-up care and medicine.

**Findings:**

All 563 participants who were enrolled in Drive-Mind-2 were screened for psychiatric disorder, and 185 of them were included in a psychiatric follow-up. The total cost of the psychiatric intervention was estimated at $88.7 per participant per year. Recurring goods and services represented 44% of total costs (including medication and transport allowance) and human resources represented 37%. The cost of one screening visit was estimated at $9.8 per participant.

**Conclusions:**

Costs for a community-based psychiatric intervention were very low in our study. This data can be used by policy makers in Vietnam to improve a mental health system that is poorly developed and expensive for the patient.

## Introduction

Co-occurrence of psychiatric disorders and substance use is common among people who use drugs (1–4). Due to scarcity of mental health settings and professionals in many parts of the world, access to psychiatric care is limited, particularly for key populations facing social vulnerability and stigma (5–8). The need for alternative interventions has been emphasized, including community-based interventions and peer-support, after skill transfer and task shifting (9–12). In other words, these interventions do not rely exclusively on psychiatrists and nurses, but also involve peers in identifying needs and designing solutions, as well as in the support itself. This type of intervention often takes place in the community rather than in conventional care settings (hospitals and health centers).

Following these recommendations, we proposed a model for a community-based psychiatric intervention among PWID in Vietnam, where access to mental health care is known to be difficult and costly (13–16). The DRIVE-Mind 1 cohort study took place for 12 months in 2019–2020 in Haiphong, Vietnam, among the participants in the larger DRIVE cohort (17). It relied on community-based organization (CBO) and psychiatrists, and showed good effectiveness on mental health outcomes (acceptability of the intervention and retention in care, marked reduction in depression, psychotic disorders and suicidal risk, significant clinical global improvement), stigma, drug use (significant heroin and alcohol use reduction) and overall quality of life (18). It was followed by a similar 12-months cohort study in 2022-2023 (DRIVE-Mind 2), that aimed to show effectiveness in reducing viral transmission and to demonstrate that psychiatric community-based intervention among PWID is sustainable over the long term.

A complementary cost-analysis of this intervention is necessary, in addition to evaluating health outcomes, to guide policy makers and advocate for alternative mental health care. Community-based interventions are rarely, if ever, subject to detailed cost analysis, even though such information is crucial for public authorities to assess their relevance and estimate the resources needed for implementation. Indeed, a 10-year literature review published in 2007 (19) found that community mental health services in low- and middle-income countries improve outcomes and may reduce costs, mirroring results from higher-income settings. Building on this evidence, more recent work by Shalaby et al. highlights the growing integration of peer support systems in mental health and addiction services, emphasizing that their success depends on strong collaboration and sustained commitment from all stakeholders (20). However, despite these advances, the evidence supporting task-sharing approaches targeting substance use and substance use disorders remains limited and debated (21), underscoring the need for further research, including detailed cost evaluations.

This prompted us to designed a micro-costing study nested in the DRIVE-Mind 2 study to estimate, from a societal perspective, the annual cost per participant for screening PWID for psychiatric disorders, and for providing them with follow-up care and medicine.

## Methods

### Psychiatric intervention design

The DRIVE-Mind 2 cohort study (NCT05886504) took place between March 2022 and May 2022 at two different community-based organization (CBO) offices, with support of a Vietnamese non-governmental organization (The Center for Supporting Community Development Initiatives, SCDI).

The PWID currently or previously diagnosed with a psychiatric disorder were proposed a 12-month community-based psychiatric and harm reduction intervention, and were compared after intervention to a control PWID free from any psychiatric diagnosis who only benefited from harm reduction intervention. All the participants of the Drive-Mind 1 survey were eligible for the enrollment in the psychiatric cohort, independently of their current psychiatric status at the inclusion visit (still symptomatic or clinically recovered). Participants initially enrolled in the control group who were later diagnosed with a psychiatric disorder during the one-year follow-up were also offered the psychiatric intervention.

Psychiatric disorders, severity of drug use and quality of life were assessed for every participant during three visits: at the start of the study, at 6 months and at 12 months. During these visits, scheduled to last six weeks, trained psychiatrists from Hai Phong school of Medicine and Pharmacy systematically administered the following modules of the Mini-International Neuropsychiatric Interview (MINI 5.0.0) (22): major depressive disorder, psychotic disorder or suicide risk. In addition, they provided their clinical input to interpret MINI findings and assessed the mental health status of every PWID to detect any other disorder requiring psychiatric care. Two versions of the clinical global improvement (CGI) scale were used to assess the initial severity of the illness and the improvement in clinical condition compared to cohort initiation. At each study site (two in total), one psychiatrist was involved in the assessment and follow-up (support, therapeutic education, prescription) of the PWID included in the cohort. In the interval between two visits, the frequency of follow-up psychiatric consultations with participants was scheduled according to the individual clinical condition of each PWID. The study psychiatrists chose among those with proven therapeutic equivalence but more affordable pricing, ensuring both treatment quality and cost-efficiency. These medications were centrally procured for the research project and provided free of charge to participants through the community-based intervention, in accordance with study protocols. Two antidepressants (sertraline and mirtazapine), four antipsychotics (risperidone, olanzapine, sulpiride, quetiapine), and melatonin for sleeping disorders were available. When needed, participants could be hospitalized in the mental health department and associated fees paid by research funds.

All CBO members were trained on psychiatric disorders and care by psychiatrists with support from SCDI staff familiar with these topics. A limited number of CBO members received additional training in Hai Phong or at National level with support from Hai Phongs’ Medical University and SCDI on motivational interviewing, psychosocial interventions, drugs and drug interactions with mental health, in order to develop group intervention for the participants and families.

All CBO members and psychiatrists tasks during the intervention are detailed in **Supplementary Table S1**.

### Data collection and cost estimation

To conduct the cost analysis, data was collected at both sites following a standardized protocol and set of tools (23–25). All resource costs related to psychiatric care for people who inject drugs (PWID) were documented in 2023 Vietnamese Dong and converted to US Dollars, regardless of funding source. Cost data were collected across four main categories: (A) staff salaries (psychiatrists and CBO members), (B) recurring goods and services (medications, transportation, and consumables), (C) hospitalization, and (D) fixed, and facility costs (rent for premises, utilities, equipment depreciation).

Given that the DRIVE-Mind 2 cohort included both mental health and harm reduction services, time allocated specifically for mental health care was estimated using two-week time records completed by CBO workers. In estimating staff costs, we accounted not only for direct contact time with participants, but also for non-clinical activities such as follow-up, participation in meetings, and organization of discussion groups. Consequently, human resource costs were calculated based on the number of half-days spent on site rather than solely on minutes of direct interaction with participants. Coordination costs and training of CBO workers were taken into account.

Study coordinators, project manager and implementing partners from the DRIVE-Mind 2 study were interviewed to gather comprehensive information on the types and amounts of human and material resources (staff salaries, rent, utilities, equipment), needed to support psychiatric care for PWID. The associated costs were then estimated using administrative records. Medications, transportation and hospitalization costs were estimated using documented feedback from both CBO members and psychiatrists. Fixed, facility and consumables-related costs were estimated using the maximum staff time spent on site as a cost driver. All equipment (office furniture, computers, printers, fingerprint machines, medicine boxes) was depreciated over 3 years.

A societal perspective was adopted to calculate the annual per-participant cost of stand-alone mental health care for PWID, by summing the costs from the four categories listed above. We did not discount the costs given the one-year time horizon of the study.

### Sensitivity analysis

To account for variability in costs across settings, we modeled two alternative scenarios for variable costs: one where all cost components were 50% less expensive than observed values, and another where all components were twice (200%) the observed costs.

Additionally, to estimate the intervention costs for populations at higher risk of psychiatric disorders, such as young people who use drugs, we simulated a scenario with a 50% prevalence of psychiatric diagnosis following screening. This increase reflects a larger number of participants requiring regular psychiatric follow-up, and we performed a separate micro-costing analysis to capture these effects.

### Ethical considerations

The DRIVE-Mind 2 research protocol was approved by the Institutional Ethics Committees of the Hai Phong Medicine and Pharmacy Faculty in Vietnam on August 30^th^, 2021. Individual written informed consent was obtained from all participants prior to participation in the cohort study.

### Role of the funding source

This work and the Drive-Mind 2 cohort study, including all costs related to the intervention, was supported by a grant from ANRS│MIE (France) (#131520). The funding agency had no role in designing the research, data analyses, or preparation of the report.

## Results

### Participant’s characteristics

Between March 2022 and April 2023, 563 participants were enrolled: 378 PWID in the control group (198 were positive for HIV), 185 in the psychiatric cohort (77 were positive for HIV). Mean age was 48.3 years and 91.3% of them were male (**Table 1**).

**Table 1:** Socio-demographic characteristics of DRIVE-Mind 2 participants at baseline.

	Total (n=563)	Control group (n=378)	Psychiatric cohort (n=185)
Age [mean (SD)]	48.3 (7.5)	50.4 (8.3)	47.2 (6.8)
Gender: male (%)	514 (91.3)	169 (91.4)	345 (91.3)
Marital status (%)			
*single*	150 (26.6)	53 (28.7)	97 (25.7)
*legally married*	241 (42.8)	59 (31.9)	182 (48.2)
*living maritally*	18 (3.2)	7 (3.8)	11 (2.9)
*separated*	143 (25.4)	61 (33.0)	82 (21.7)
*widowed*	11 (2.0)	5 (2.7)	6 (1.6)
Having a health insurance (%)	409 (72.7)	120 (64.9)	289 (76.5)
Arrested in the last 6 months (%)	13 (2.3)	6 (3.2)	7 (1.9)
Source of income			
*regular salary*	150 (26.6)	36 (19.5)	114 (30.2)
*temporary work/ self-employment*	263 (46.7)	82 (44.3)	181 (47.9)
support from family/relatives	240 (42.6)	94 (50.8)	146 (38.6)
*no income*	6 (1.4)	2 (0.8)	4 (2.6)
Current major depressive episode (%)	–	–	76 (41.1)
Current psychotic disorder (%)	–	–	46 (24.9)
Suicide risk (low, intermediate or high) (%)	–	–	59 (31.9)
HIV positive serology	275 (48.8)	198 (52.4)	77 (41.6)

**Figure 1:**
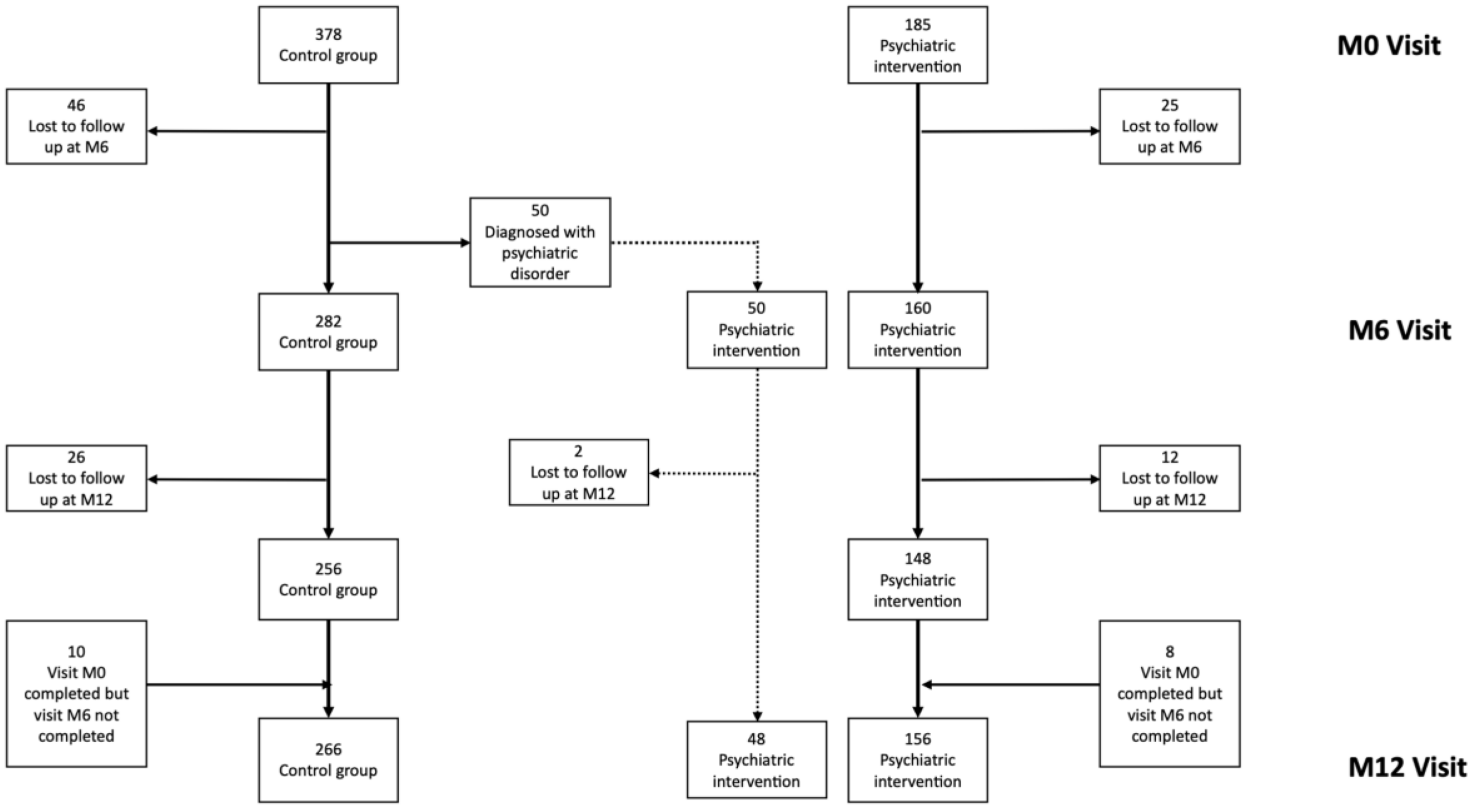
Flow chart for the DRIVE-Mind 2 intervention.

At M0 visit, half of the 185 participants in the psychiatric cohort were newly screened positive for one or more current psychiatric disorders. The remaining participants had been previously diagnosed during the Drive-Mind 1 study but showed no current symptoms (due to a psychiatric follow-up and treatment that was still ongoing, or to natural remission). During M6 visit, 50 PWID from the control group were diagnosed with a current psychiatric condition requiring intervention and joined the psychiatric cohort (**Figure 1**).

Retention rate in the psychiatric cohort was high with 156/185 (84%) PWID coming back at M12 visit (**Figure 1**). At cohort initiation, depression was the most frequent current psychiatric disorder, affecting 40% of the intervention group, followed by psychotic disorder (affecting 25%). All measured psychiatric disorders significantly decreased between M0 and M12. Among those showing a psychiatric disorder, 132 (85%) were very much or much clinically improved at M12 visit (using CGI scale), 13 (8%) minimally improved, 11 (7%) unchanged or worsen.

### Activities and staff

Over 12 months, 1,425 screening visits and approximately 1,420 psychiatric follow-up consultations were conducted at CBO offices for participants with psychiatric disorders. Staffing varied by intervention phase: during the three screening periods (M0, M6, M12), each site required one full-time (i.e. ten half-days per week) psychiatrist and five half-time (i.e. five half-days per week) CBO members for six weeks; during the 40-week follow-up phase, staffing dropped to 0.1 full time equivalent (i.e. one half-day per week) psychiatrist and five half-time (i.e. five half-days per week) CBO members. No differences were noted between sites. Human resource costs, i.e. staff salaries were split between screening (all participants) and follow-up (psychiatric cohort only). Screening involved 4 to 21 participants per day (average 8.7), while follow-up included an average of 11.7 consultations per participant, each lasting between 2 and 12 minutes, with frequency depending on clinical needs. The average cost of staff salaries per participant was $4.7 for a screening visit, and $59.8 for psychiatric follow-up, with CBO members responsible for 82% of the follow-up costs (**Table 2**).

**Table 2:** Annual cost of psychiatric screening and follow-up among DRIVE-Mind 2 participants.

	Average cost of the entire intervention per participant (all participants)	Average cost of a screening visit per participant (all participants)	Average follow-up cost per participant diagnosed with psychiatric disorder
Human resources (staff salaries)	$32.5	$4.7	$59.8
Fixed and facility costs*	$15.6	$1.9	$31.8
Recurring goods and services†	$39.4	$3.2	$97.6
*Medication*	$16.8	–	$53.1
*Transportation allowance*	$17.6	$3.1	$28.8
Hospitalization costs	$1.2	–	$3.8
Total costs	$88.7	$9.8	$193.0

*Fixed and facility costs include rent, utilities, equipment.

†Recurring goods and services include medication, transportation allowance, and consumables.

### Recurring goods and services (psychiatric medication, transportation allowance, and consumables)

Transportation allowance costs were estimated at an average of $3.1 per participant for a round trip from home to study site, either for a scheduled screening visit or as part of psychiatric follow-up, leading to an average transportation allowance of $28.8 per participant diagnosed with a psychiatric disorder. The average cost of medication was estimated at $7.1 per treated participant per month, amounting to a total of $53.1 per participant diagnosed with a psychiatric disorder (**Table 2**). Consumables accounted for less than $0.5 per participant.

### Hospitalization

Two one-month hospitalizations in a mental health hospital were reported over the course of one year within the psychiatric cohort, with an estimated total cost of $666.7 – equivalent to $1.2 per participant per year, all participants (**Table 2**).

### Fixed and facility cost

Fixed and facility costs were estimated at $15.6 per participant per year (across all participants), accounting for 17.5% of total costs. These costs represented a very small portion of costs during screening visits, which lasted only six weeks each with rent being one of the main items of costs in this cost category (**Table 2**).

### Overall cost of the psychiatric intervention

**Figure 2:**
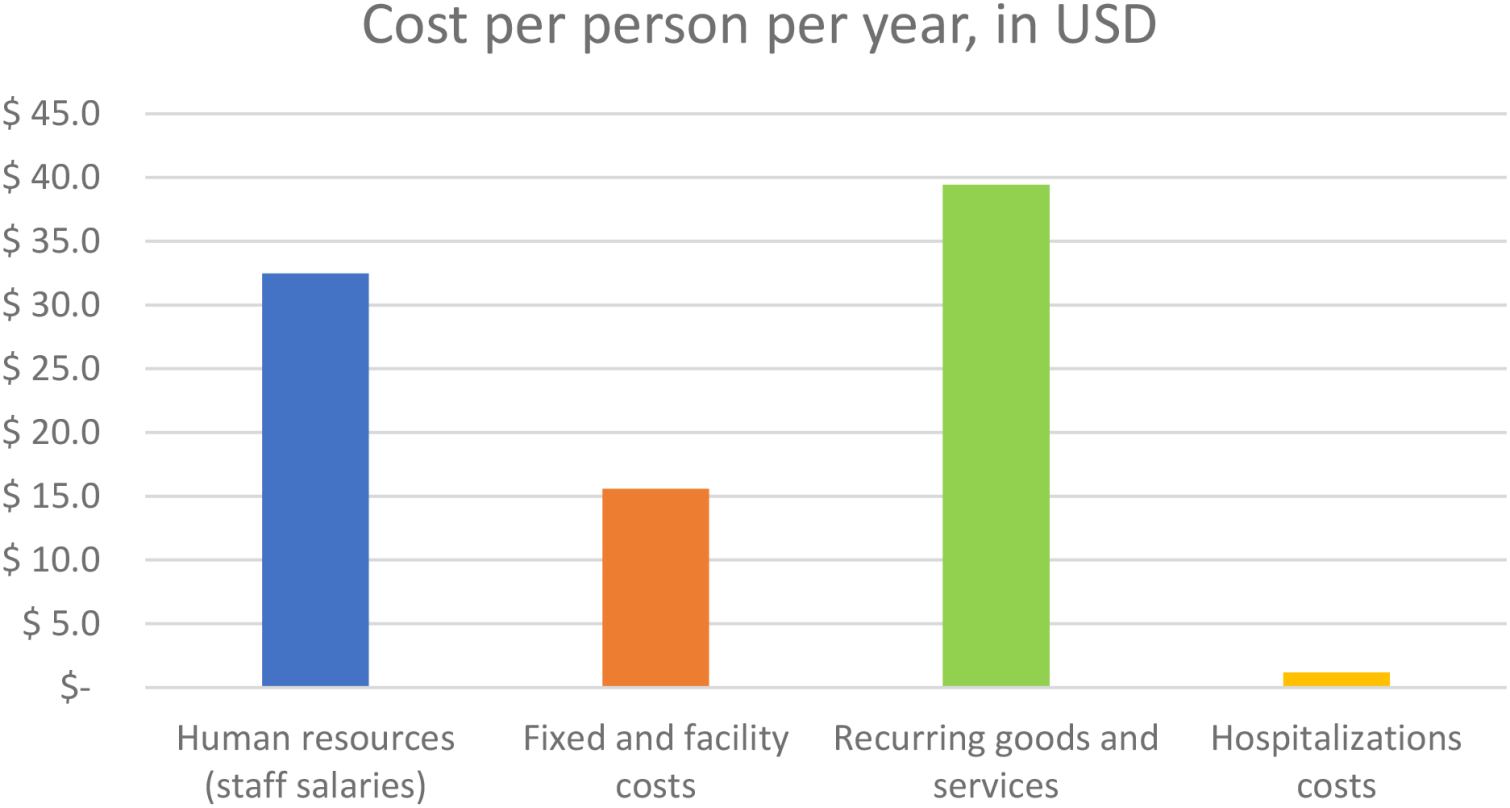
Average cost of the entire Drive Mind 2 intervention, per participant per year, in US dollars.

The total cost of the psychiatric intervention was estimated at $88.7 per participant per year (**Table 2**), including all participants regardless of psychiatric status. The majority of costs came from recurring goods and services - mainly medication and transportation allowance- amounting to $39.4 (44% of total costs), followed by staff salaries at $32.5 (37% of total costs) (**Figure 2**). The cost of a single screening visit was estimated at $9.8 per participant.

Focusing on the 185 persons in the psychiatric cohort, the total cost of one year of psychiatric follow-up was estimated at $193 per participant. Transportation and medication remained the largest cost components during follow-up, accounting for half of the total cost.

### Sensitivity analysis

**Figure 3:**
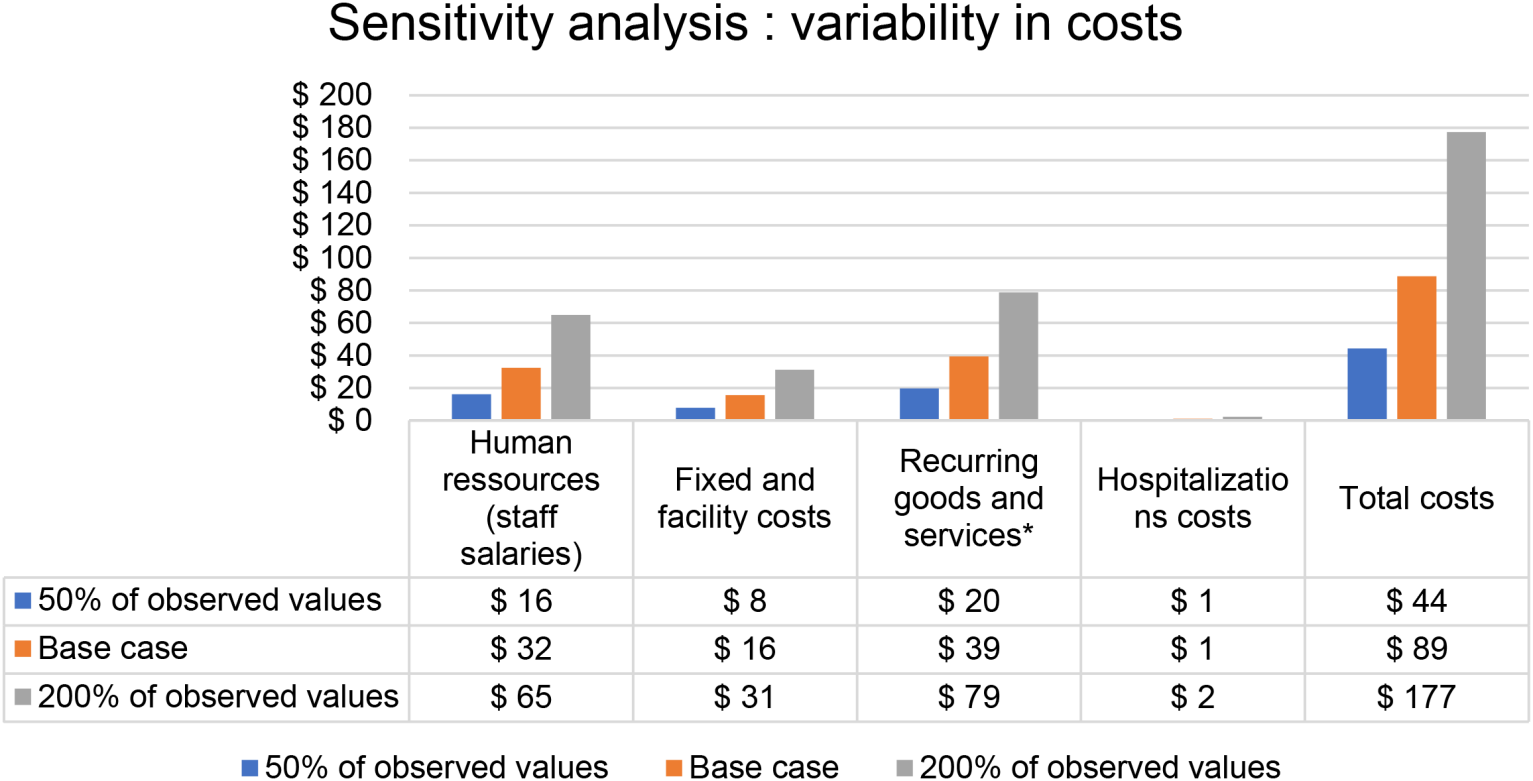
Sensitivity analysis modeling two alternative scenarios for cost components: all components at 50% and 200% of observed values (base case).

Variability in costs is shown in **Figure 3**. Overall costs range from $44.3 (-50% of observed values) to $177.4 (200% of observed values). In the simulation assuming a 50% prevalence of current psychiatric disorder (compared with 33% in our population), fixed and facility costs as well as overall follow-up costs, were reduced as expected, while the cost per participant increased moderately (from $88 to $114) (**Table 3**). These results show the low cost of the intervention even among population most at risk of developing psychiatric disorders such as young people who use drugs.

**Table 3:** Annual cost of psychiatric screening and follow-up among DRIVE-Mind 2 participants - simulation assuming a 50% prevalence of psychiatric disorder.

	Average cost of the entire intervention per participant (all participants)	Average cost of a screening visit per participant (all participants)	Average follow-up cost per participant diagnosed with psychiatric disorder
Human resources	$42.0	$4.7	$60.0
Fixed and facility costs*	$15.6	$1.9	$20.9
Recurring goods and services†	$55.1	$3.2	$96.9
*Medication*	$25.4	–	$52.8
*Transportation allowance*	$22.2	$3.1	$28.7
Hospitalization costs	$1.8	–	$3.9
Total costs	$114.5	$9.8	$181.7

*Fixed and facility costs include rent, utilities, equipment.

†Recurring goods and services include medication, transportation allowance, and consumables.

## Discussion

With a total cost of $88 per person per year- including transportation costs - the DRIVE-Mind 2 intervention proved to be low cost considering its substantial benefits on health outcomes. Furthermore, this study was conducted from a societal perspective, accounting for all costs borne by health insurance and beneficiaries.

Beyond individual-level benefits, improved mental health among PWID contributes to reduced viral transmission within the population, fewer hospital admissions for non-psychiatric conditions, and overall cost savings for the health system (26).

To our knowledge, this is the first micro-costing analysis study of a psychiatric community-based intervention performed in Vietnam, or even in South Asia. Nevertheless, the intervention cost can be compared to that of outpatient psychiatric care in Haiphong. According to field workers and psychiatrists interviewed in our study, a single “on demand” psychiatric consultation in the current mental health care system lasts from 5 to 60 minutes and costs from $12 to $89 in Haiphong depending on the setting (hospital vs private clinic), and without taking into account transportation and medication costs. Costs for “on-demand” consultations in the hospital are usually lower (roughly $12 to $20) but patients are often prescribed costly additional laboratory tests. In Vietnam the cost of medication for patients varies depending on the type of disorder, its severity, and the patient’s insurance status, but it generally remains high. For instance, patients with depression without insurance generally incur out-of-pocket expenses ranging from $35 to $103 per month (27). Medication costs in our study were significantly lower than those in regular clinical settings, primarily because the drugs used were selected from a short list of essential, evidence-based psychotropic medications. The lower consultation costs can be explained by effective partnerships with psychiatrists and remuneration that is not based on the number of additional tests performed.

Integrating mental healthcare into methadone maintenance treatment clinics or HIV clinics has long been recommended (28–30) and could further reduce costs (31), by reducing fixed costs and transportation costs (accounting for 37% of overall costs in our study). It is worth noting that in Vietnam, primary healthcare facilities (commune and district health centers) generally lack specialized psychiatric consultation rooms and psychiatrists on staff. Individuals needing mental health services typically must seek care at psychiatric hospitals or psychiatric departments within general hospitals. Although the Community Mental Health Care model relies on non-specialists at the community-level, the lack of professional facilities at the grassroots level remains a significant barrier (16). Strengthening infrastructure and personnel at commune health centers could improve accessibility and reduce the burden on higher-level hospitals. The high retention rate in our psychiatric cohort supports the feasibility of this low-cost community-based model, represents a good alternative for delivering services and suggests strong acceptance by PWID – a group traditionally underserved and stigmatized in institutional settings.

There are several limitations to our study. The study population is very specific - PWID living in a dense urban area - and we can only assume that costs calculation would be similar in other key populations. Implementing the intervention in rural settings, for example, could result in significantly higher transportation costs. Nevertheless, the intervention was carried out among one of the most hard-to-reach populations, requiring substantial human resources from CBO members to ensure a high level of linkage to care. Moreover, our sensitivity analysis indicates that even with a twofold increase in all cost components, the overall cost of the intervention remains modest when compared to the current expenses for psychiatric consultations and medications within the existing mental health care system (as previously discussed). Only three psychiatric conditions were investigated in this study, whereas data from the literature also highlight the high prevalence in this population of personality, anxiety and post-traumatic stress disorders. This choice reflected the need to first identify conditions requiring rapid therapeutic intervention. It is possible that this choice influenced the estimated cost of care, although, as mentioned in the sensitivity analysis, adjusting for a higher prevalence of disorders would have little impact on the overall estimate. A common limitation of micro-costing studies, which also applies to the present analysis, is that cost estimates are based on a limited time horizon—in this case, one year—and may therefore not capture potential cost variations over the longer term. Nonetheless, this limitation is mitigated by the relevance of the micro-costing approach in the context of DRIVE-Mind 2, an innovative intervention targeting a highly marginalized and understudied population – PWID -. In the absence of standard cost data, micro-costing enables a detailed and context-specific assessment of actual resource use. Lastly, the cost associated with the partnership between the university and the CBOs is always difficult to quantify precisely, but most of this cost (the training of CBO workers) has been taken into account in our study.

In conclusion, our study highlights the low cost and operational feasibility of a community-based psychiatric intervention model for PWID. These findings can support advocacy efforts with policymakers to scale up mental health services, which are currently limited, costly, and inadequately accessible, especially for marginalized populations. The results may also serve as a foundation for future cost-utility analyses. Moving forward, research should focus on assessing the long-term sustainability of this model and its potential integration into Vietnam’s public health and insurance systems, particularly after donor funding ends.

## Data Availability

The raw data supporting the conclusions of this article will be made available by the authors, without undue reservation.
