# Supplementary material for: Micro-costing analysis of a community-based psychiatric intervention among people who inject drugs in Haiphong, Vietnam
Source: Front Psychiatry. 2025 Dec 4;16:1676340. doi: 10.3389/fpsyt.2025.1676340 (PMC12712603; doi:10.3389/fpsyt.2025.1676340)
Supplement: Supplementary file 1 [file Table1.pdf]

Supplementary table 1: CBO and psychiatrist tasks during the intervention

CBO tasks related to mental health	• Individual and group information, education and communication on mental health, mental disorders, their treatments, side effects of the treatments and time to action, adherence to treatment<br>• Distribution of flyers on harm reduction for methamphetamine users (including psychiatric consequences) and on mental health for peers and their family<br>• Recall appointments with psychiatrists and payment for transportation fees<br>• Information to psychiatrists in case of unusual events or worrying situation<br>• Offer of closer follow-up for participants signaled by the psychiatrist and when possible, contact the family<br>• Collection of information on participants lost for follow-up or in case of poor adherence<br>• Referral of severe cases to hospital and payment for hospitalization fees when necessary<br>• Meetings with family to inform, support and educate when necessary<br>• Link between family and doctors.
Other CBO tasks	• Linkage to HIV care and methadone maintenance treatment<br>• Administrative support (health insurance, identity card, resident card)<br>• Harm reduction intervention (counselling, clean needles-syringes and condoms distribution)<br>• Collection of data on drug use, sexual behaviors and use of drug-related facilities during face-to-face structured interviews
Psychiatrists from the mental health department	• Psychiatric consultations on CBO site<br>• Prescription on CBO site<br>• Delivery of treatment by psychiatrists on CBO site<br>• Coordination of the follow-up
